# Identification of novel microtubule inhibitors effective in fission yeast and human cells and their effects on breast cancer cell lines

**DOI:** 10.1098/rsob.210161

**Published:** 2021-09-08

**Authors:** Jun Morishita, Paul Nurse

**Affiliations:** ^1^ Laboratory of Yeast Genetics and Cell Biology, Rockefeller University, New York, NY 10065, USA; ^2^ The Francis Crick Institute, London NW1 1AT, UK

**Keywords:** fission yeast, cancer, tubulin, colchicine-binding agents

## Abstract

Microtubules are critical for a variety of cellular processes such as chromosome segregation, intracellular transport and cell shape. Drugs against microtubules have been widely used in cancer chemotherapies, though the acquisition of drug resistance has been a significant issue for their use. To identify novel small molecules that inhibit microtubule organization, we conducted sequential phenotypic screening of fission yeast and human cells. From a library of diverse 10 371 chemicals, we identified 11 compounds that inhibit proper mitotic progression both in fission yeast and in HeLa cells. An *in vitro* assay revealed that five of these compounds are strong inhibitors of tubulin polymerization. These compounds directly bind tubulin and destabilize the structures of tubulin dimers. We showed that one of the compounds, L1, binds to the colchicine-binding site of microtubules and exhibits a preferential potency against a panel of human breast cancer cell lines compared with a control non-cancer cell line. In addition, L1 overcomes cellular drug resistance mediated by βIII tubulin overexpression and has a strong synergistic effect when combined with the Plk1 inhibitor BI2536. Thus, we have established an economically effective drug screening strategy to target mitosis and microtubules, and have identified a candidate compound for cancer chemotherapy.

## Graphical abstract

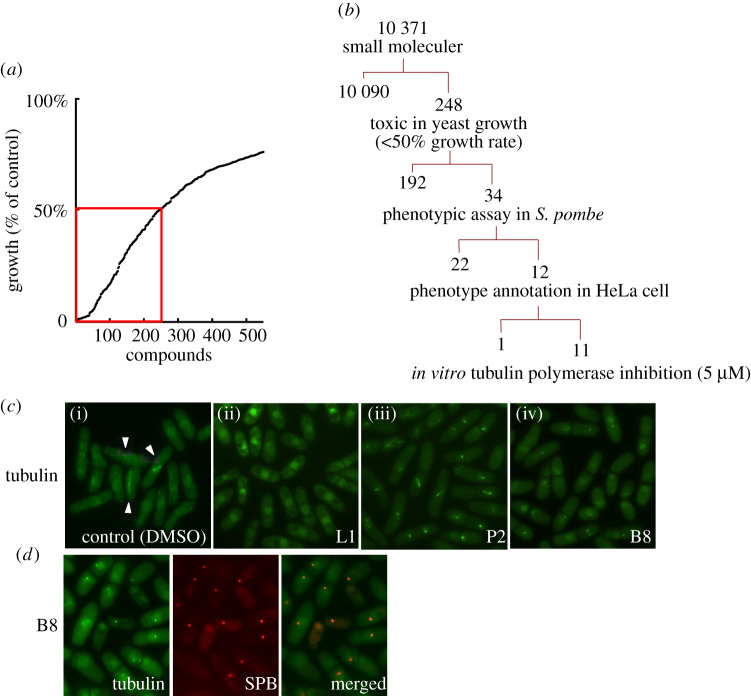

## 1. Introduction

Microtubules are dynamic protein polymers composed of α- and β-tubulins. They are found in eukaryotic cells where they form cytoskeletal architectures supporting a wide range of cellular functions, such as chromosome segregation, cell shape, intracellular transport and cellular movement [1,2]. The organization of microtubules changes dramatically during the cell cycle: in interphase, microtubules form arrays throughout the cytoplasm and are relatively stable, while during mitosis they form a bipolar mitotic spindle and become highly dynamic, with a 20–100-fold increase in microtubule dynamics. When proliferating cells are exposed to microtubule inhibitors, bipolar spindle formation and microtubule attachment to kinetochores are inhibited [3], activating the spindle assembly checkpoint (SAC). This causes cell cycle arrest prior to the metaphase–anaphase transition [4], eventually leading to apoptosis in tissue culture cells [5].

Anti-microtubule drugs such as colchicine, nocodazole (NOC), benomyl and paclitaxel are commonly used to study the diverse functions of microtubules and to synchronize cells at mitosis. Drugs that both stabilize microtubules (e.g. taxanes such as paclitaxel) and destabilize microtubules (e.g. vinca alkaloids, such as vinblastine) are also used as anti-cancer agents [6,7]. Most anti-microtubule drugs bind one of four main sites within microtubules impacting tubulin stability: the laulimalide site (stabilizing), taxane/epithilone site (stabilizing), colchicine site (destabilizing) or vinca alkaloid site (destabilizing) [8]. Among these drugs, taxanes have been successfully used in the treatment of solid tumours, particularly in ovarian and breast cancers, for over 25 years [9]. However, their clinical utility can be limited by side effects such as peripheral neuropathy and neutropenia, and by the development of drug resistance. Resistance to taxanes and other anti-microtubular drugs eventually develops in more than half of cancer patients through different mechanisms [10,11].

In this paper, we aimed to identify new anti-microtubule drugs, with the objectives of generating new reagents for the physiological investigation of microtubular functions in cells and also for the identification of new lead drugs that could be used in cancer therapy. To search for new low-molecular-weight compounds with anti-microtubular activity, we used the fission yeast, *Schizosaccharomyces pombe*, a single-cell eukaryote, that allows a rapid and economic workflow for screening and identifying the molecular targets of drugs. We executed a high-throughput phenotype-based screen for small molecules that inhibit fission yeast growth and then tested their effects on microtubules. Candidate molecules were then further tested for their ability both to disrupt microtubules in human cells and to specifically inhibit the growth of human breast cancer cell lines. These novel small molecules inhibit mitotic spindle assembly effectively in both fission yeast and human cells, and provide potential drug candidates for cancer therapies.

## 2. Results

### 2.1. Screening of compounds that disrupt mitosis

**Figure 1.**
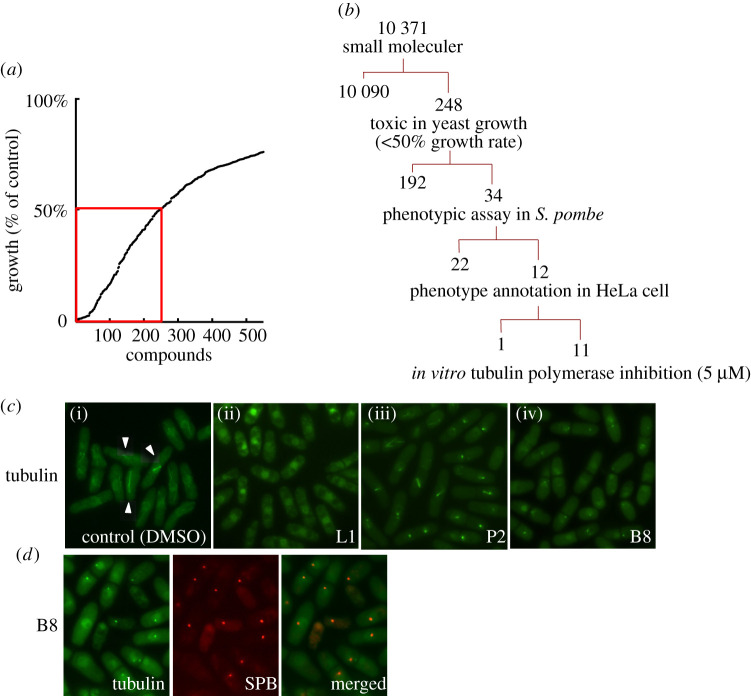
Phenotypic screening in MDR-sup fission yeast (*a*). Growth assay screening using MDR-sup strain. The optical density was measured with 20 µM of each compound and 248 compounds that gave at least 50% growth inhibition were used for further phenotyping marked by the red lines. Growth was normalized to DMSO-treated cells. (*b*) Schematic representation of the screening workflow. (*c*) Examples of the phenotypic assay using L1, P2 and B8 in the MDR-sup strain expressing GFP-atb2 (tubulin) are shown. Cells were incubated in 5 µM compound for 3 h and atb2 signals were detected in living cells. (i) Cells in absence of any compound. In interphase cells, *α*-tubulin Atb2 protein was detected in the nucleus and interphase microtubules, while mitotic spindles were detected in mitotic cells (arrows). (ii) In the presence of L1, microtubules were depolymerized and tubulin signals were detected distributed throughout the nucleus. (iii) In the presence of P2, short spindles were detected but anaphase spindles were not. (iv) In the presence of B8, interphase microtubules were not detected, while, dots were detected. (*d*) Tubulin (atb2-GFP) and SPB (sid4-mcherry) were imaged. Some dots produced by incubating with B8 were co-localized with SPB.

We have previously screened a collection of 10 371 small molecules to identify compounds that inhibit the growth of a multiple-drug-sensitive fission yeast strain (MDR-sup) [12,13]. We selected 248 compounds (2.4% of the total) that inhibited fission yeast cell growth rate by at least 50% (figure 1*a*) and subsequently conducted a phenotypic screen of these to identify drugs that affect microtubule organization (figure 1*b*). After the MDR-sup cells were treated with 5 µM of each compound for 3 h, microtubules were monitored using GFP tagged α-tubulin Atb2 and live-cell fluorescence microscopy. In untreated interphase cells, microtubules form long cytoplasmic arrays that run parallel to the long cellular axis, and in mitotic cells, microtubules form a bipolar spindle in the nucleus and the cytoplasmic microtubules disappear (figure 1*c*(i), arrow). Tubulin is present in the nucleus in interphase cells but does not form detectable nuclear polymer structures. This screen identified 34 compounds that induced cytological phenotypes indicative of defects in microtubule organization or function. The observed defects could be grouped into three classes: (i) microtubules are undetectable, and α-tubulin accumulates in the nucleus (as seen with compound L1 in figure 1*c*(ii)); (ii) mitotic cells exhibit only short bipolar spindles (as seen with compound P2 in figure 1*c*(iii)); and (iii) tubulin is found as a single dot at the SPB without the formation of short or long microtubules (as seen with compound B8 in figure 1*c*(iv) and figure 1*d*).

**Figure 2.**
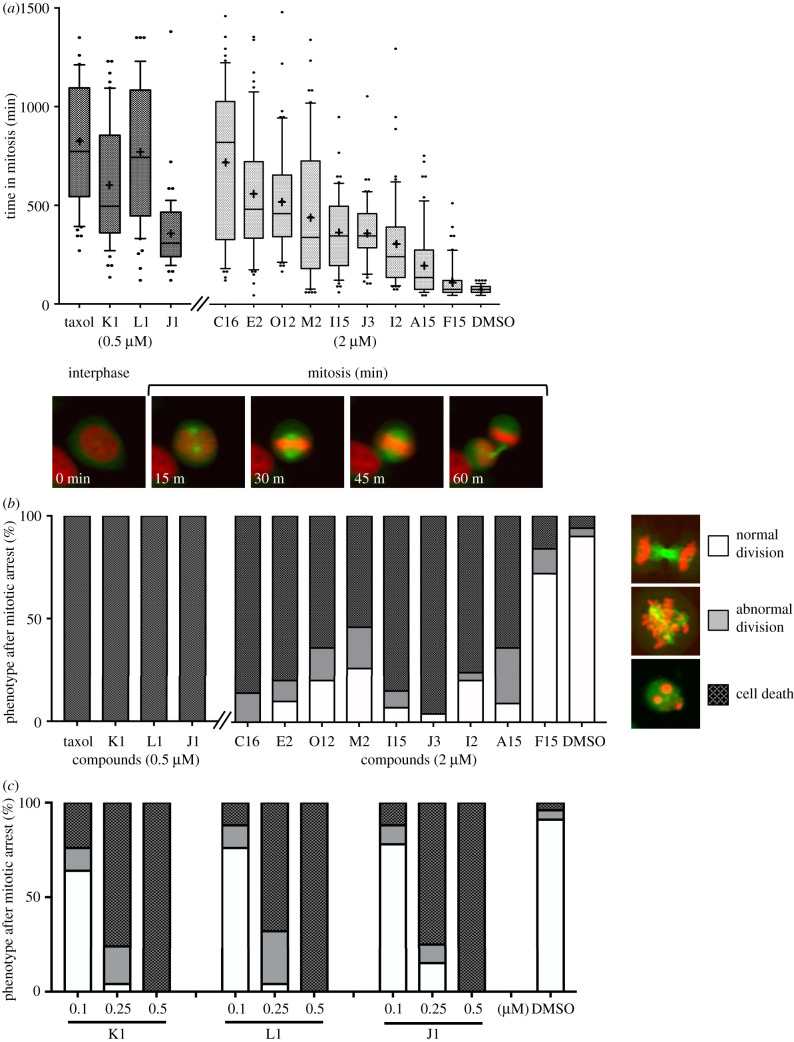
Effect of compounds on mitotic progression in HeLa cell (*a*). Duration of mitosis was analysed by live imaging of HeLa cells stably expressing H2B-mcherry (red) and EGFP-a tubulin (green) treated in the presence of 0.5 µM (K1, L1, J1) or 2 µM (C16, E2, O12, M2, I15, J3, I2, A15, F15) compound for 24 h and the time in mitosis was measured in 200 cells. DMSO-treated cells are shown, which served as the vehicle control. Show mean as ‘+’. (*b*). The Cell fate after mitosis (normal division, abnormal division or cell death) was quantitatively assessed after the mitotic arrest observed in (*a*). (*c*) Dose-dependent change of phenotypes after mitotic arrest with K1, L1 and J1.

To determine whether these 34 compounds also induce mitotic abnormalities in human cells, a second screen of these compounds was conducted using HeLa cells. We used time-lapse microscopy to monitor mitotic progression in HeLa cells expressing mCherry-tagged histone H2B to mark chromatin, and EGFP-tagged α-tubulin to monitor microtubules over a 24 h time period in the presence of 2 µM of each of the 34 compounds. The duration of mitosis was determined by live-cell imaging, defined as the period from prophase to cytokinesis based on chromatin and microtubule morphologies (figure 2*a*). The control DMSO-treated cells progressed through mitosis with a mean mitotic duration of 77 ± 6 min. Among the 34 compounds we tested, nine caused a significant increase in mitotic duration in human cells (figure 2*a*), ranging from 122 ± 42 min (F15) to 720 ± 53 min (C16). Most of these compounds resulted in cells arresting in early mitosis, followed by abnormal asymmetric division and/or cell death (figure 2*b*). Cells treated with the four compounds K1, L1, J1 and B8 immediately induced cell death independent of the phase of the cell cycle, but at a lower concentration (0.5 µM), these compounds arrested cells initially in mitosis, followed by cell death (figure 2*a,b*). As described in the Discussion, we have not carried out a detailed analysis for B8 in this study. To determine whether these cell deaths were caused by apoptosis, caspase activity was assayed for 24 h using time-lapse microscopy in the presence of the 12 compounds. Caspase 3 and Caspase 7 activities were observed 12–24 h after the addition of the compounds (electronic supplementary material, figure S1A). There were also other indications of apoptosis including the morphological chances reminiscing of membrane blebbing, chromatin condensation and cell explosion (electronic supplementary material, figure S1B). Caspase-mediated apoptosis induction is similar to the effects of mitotic arrest caused by anti-microtubule drugs in HeLa cells [14,15]. At lower concentrations of 0.1 and 0.25 µM, K1, L1 and J1 induced abnormal multipolar, asymmetric cell divisions, generating 3–4 daughter cells (figure 2*c*). We conclude that these 12 compounds cause mitotic arrest followed by cell death through apoptotic activation.

### 2.2. Compounds directly inhibit tubulin polymerization and microtubule dynamics

**Figure 3.**
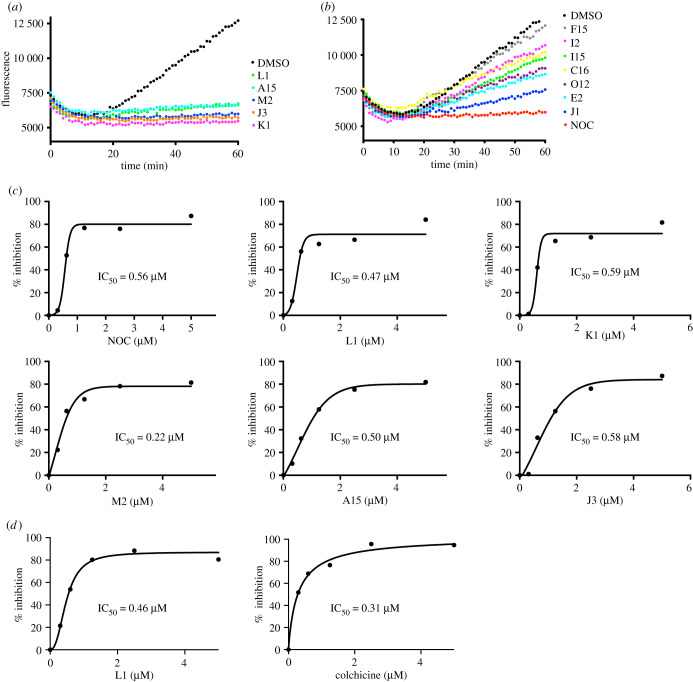
Effects of the compounds on microtubule polymerization *in vitro*. NOC inhibited tubulin polymerization in the cell-free system. Tubulin was incubated with 5 µM of the compounds and DMSO for 60 min at 37°C. The fluorescence increase was monitored every minute at 340 nm. (*a*) Compounds which abolish microtubule polymerization at 5 µM. (*b*). Compounds which inhibit polymerization activity partially at 5 µM. Nocodazole (NOC) was used as the control. (*c*). Concentration-dependent inhibition of tubulin polymerization by the compounds L1, K1, M2, A15 and J3. The IC_50_ values of each compound were generated by Prism V9 GraphPad and are indicated in the graph. (*d*) The same experiment as (*c*) but carried out at a different time. The IC_50_ values of L1 and colchicine were measured.

To determine whether the 12 compounds affected mitotic spindle organization by directly interfering with tubulin polymerization, we performed *in vitro* tubulin polymerization assays. Microtubule polymerization was induced with bovine brain tubulin (greater than 99% pure) in the presence of the compounds at 5 µM at 37°C (dotted lines in figure 3*b*) with NOC as a positive control. Tubulin polymerization was monitored every 1 min by measuring fluorescence absorbance at 340 nm for 1 h. As shown in the representative traces shown in figure 3, 11 of the 12 compounds destabilized microtubules. Five compounds showed almost complete inhibition of tubulin polymerization (figure 3*a*), and six compounds inhibited polymerization partially (figure 3*b*). The five most potent compounds inhibited *in vitro* tubulin polymerization in a concentration-dependent manner, with a similar dose dependency to NOC (figure 3*c*). While NOC had an IC_50_ of 0.56 µM, the five potent compounds had comparable IC_50s_ of K1; 0.59 µM, L1; 0.47 µM, M2; 0.22 µM, A15: 0.50 µM, J3; 0.58 µM (figure 3*c*). These results established that these five compounds possess strong *in vitro* anti-tubulin polymerization activity, and that the other six compounds have partial inhibitory effects on tubulin polymerization. The 12th compound, F15 had no observable effects on polymerization at 5 µM and therefore appears to interfere with progression through mitosis by a mechanism not involving microtubule polymerization dynamics. F15 is also the least potent of the tested compounds for both mitotic arrest and cell death.

### 2.3. Compounds alter the stability of the tubulin structure

**Figure 4.**
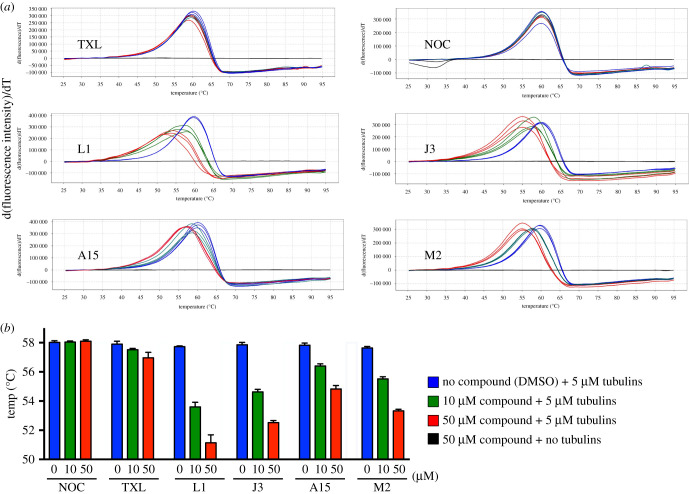
Inhibitors binding to tubulin, as probed via DSF. (*a*) Thermal unfolding of tubulin is monitored using SYPRO Orange. Five microlitres of purified tubulins are assessed in the absence of compound (blue) or in the presence of 10 µM (green) and 50 µM compound (red) respectively. DSF experiment for compounds showing a decrease in thermal stability with increase Tm shift between the native tubulin protein and tubulin protein-ligand complex. Three replicates of each condition were shown. TXL, paclitaxel; NOC, nocodazole. (*b*) The Tm values of the three replicates experiments shown in (*a*) are presented.

To determine if the compounds directly bound *α*/β-tubulin dimers, the effects of the most active inhibitors (compounds L1, J3, A15 and M2) on the stability of purified tubulins were assessed by differential scanning fluorimetry (DSF) analysis [16]. Because the compounds K1 and L1 were structural analogues, we omitted K1 from this analysis. We monitored the thermal stability of purified tubulin at 5 µM (0.27 mg ml^−1^) by gradually increasing the temperature from 25°C to 95°C in the presence of the excess Sypro orange fluorescent dye [16–18]. Fluorescence increases as the proteins denature and expose the hydrophobic residues that bind the fluorescent dye. Using this method, the melting temperature Tm of native tubulin was determined to be 57.8°C in the absence of any inhibitor (figure 4*a* blue line, B blue bar). Upon addition of an increasing concentration of the inhibitors, the unfolding transition of tubulin was shifted to lower temperatures in a dose-dependent manner. This is because the inhibitors bind and destabilize the structure of the α/β-tubulin dimers at lower temperatures (figure 4*a*, B 10 µM compound in green, 50 µM in red). By titrating the compound concentrations, we found that compounds L1, J3, A15 and M2 exhibited potent destabilizing effects with Tm ranging from 51°C to 55°C when 50 µM of the compounds was used, and from 53.5°C to 56.5°C when 10 µM was used. These effects were greater than either NOC or taxane (figure 4*b*).

**Figure 5.**
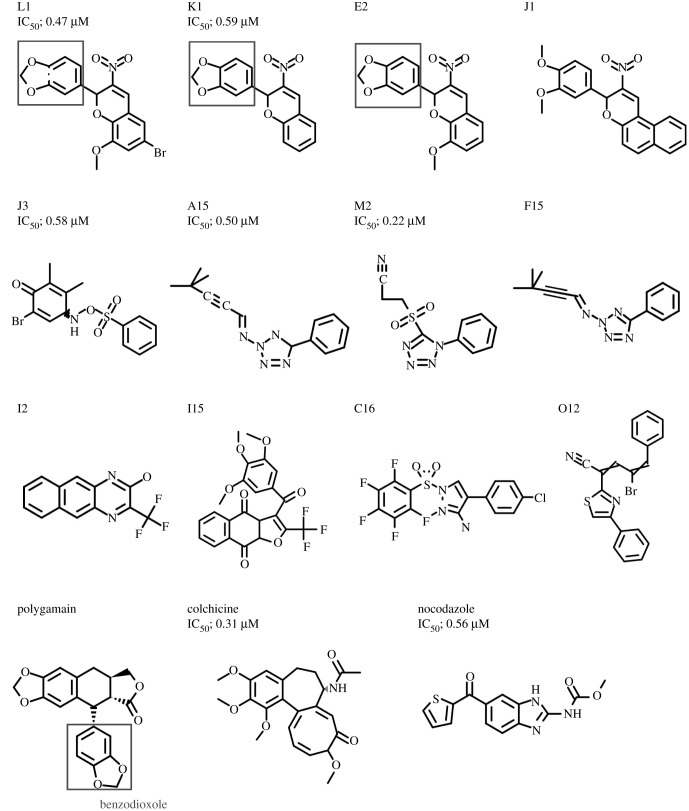
Chemical structures of tubulin inhibitors screened in this study. The IC_50_ values are indicated for the most potent five compounds as well as nocodazole and colchicine in *in vitro* assay (figure 3*c,d*). They are represented by singleton chemical structures, and four compounds (L1, K1, E2 and J1) are related structures. L1, K1 and E2 contain benzodioxole (shown in red box) which has been found in polygamain.

The chemical structures of the most potent compounds L1, K1, J3, A15 and M2 are shown in figure 5*a*. The compounds L1, K1, E2 and J1 all have structural similarities. L1, K1 and E2 have a common benzodioxole, which is also a part of polygamain (figure 5*b*), a known microtubule-depolymerizing lignan [19], whereas J1 contains a dimethoxyphenyl moiety, which shows a structural similarity to a benzodioxole. In this group, L1 possessed the strongest anti-tubulin polymerization activity with an IC_50_ of 0.47 µM, comparable to NOC with an IC_50_ of 0.56 µM (figure 3*c*). All microtubule destabilizing compounds screened in this study showed a similar phenotype in fission yeast: no interphase microtubules or mitotic spindles were detectable, and tubulin was found to be present in the nucleus (figure 1*c,b*). The same phenotype was also observed when fission yeast MDR-sup was treated with other known inhibitors of microtubule polymerization such as NOC and benomyl (electronic supplementary material, figure S2). The drug NOC that inhibits microtubule assembly [20] was hypersensitive to L1, and the cold-sensitive α-tubulin mutant *nda2-KM52* and the β-tubulin mutant *nda3-KM311* were both hypersensitive to L1 at the permissive temperature of 30°C. No such drug hypersensitivity was observed in the cell cycle mutant *cdc25-22*, condensin mutant *cut14-208* or actin mutant *act1-48* (electronic supplementary material, figure S3). Altogether, our screen has successfully identified novel direct inhibitors of microtubule polymerization in both fission yeast and HeLa cells.

### 2.4. Compound L1 displaces colchicine from tubulin

**Figure 6.**
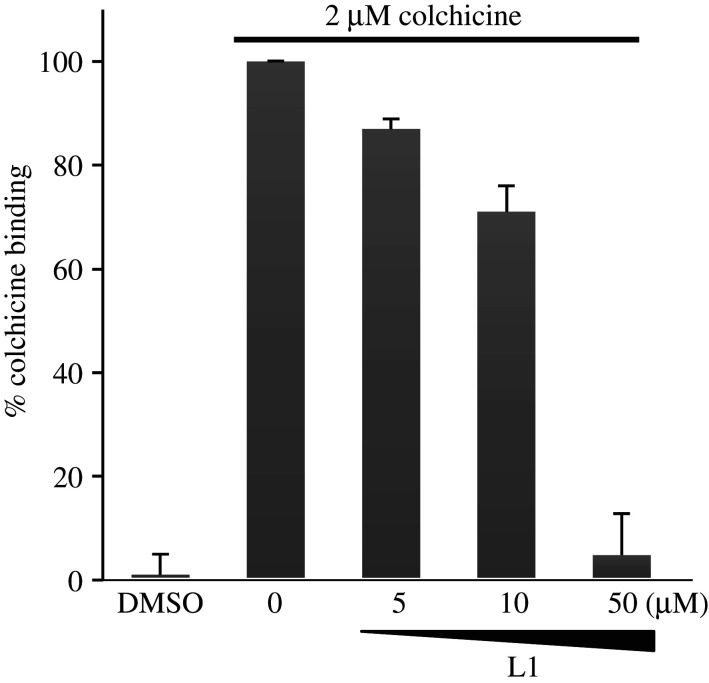
Compound L1 binds to the cholchicine binding site on tubulin L1 displaces colchicine from tubulin. Two microlitres of tubulin alone (DMSO control), 0, 5, 10, 50 µM L1, was added to 2 µM tubulin and 2 µM Colchicine, respectively. The fluorescence measured with 2 µM tubulin was set at 0%, and 2 µM tubulin and 2 µM colchicine was set 100%. Each value represents the mean ± s.e.m. of three independent experiments.

Since polygamain binds to tubulin within the colchicine-binding site, we conducted a fluorescent-based colchicine displacement assay to evaluate the ability of L1 to inhibit colchicine binding to tubulin [21] (figure 6). L1 competed with colchicine for binding on tubulin and caused a dose-dependent decrease in the fluorescence of the colchicine-tubulin complex. Five micromolar L1 inhibited fluorescence to 87%, 10 µM L1 inhibited fluorescence to 71%, and 50 µM L1 abolished the interaction between colchicine and tubulin complex completely (figure 6). These data indicate that L1 competes with colchicine binding to purified tubulin, suggesting that L1 binds to tubulin within the colchicine-binding site.

### 2.5. βIII-mediated drug resistance

**Figure 7.**
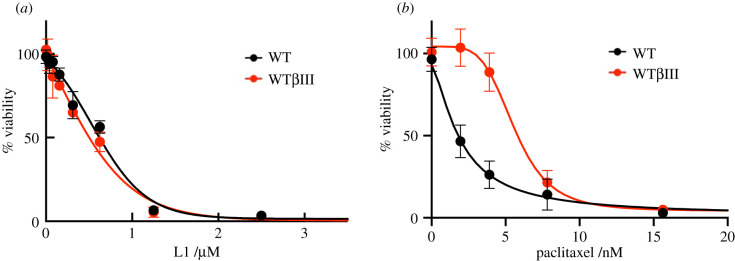
Compound L1 overcomes βIII-mediated drug resistance. L1 (*a*) and Paclitaxel (*b*) were added at increasing concentrations to HeLa cells (black line) and Hela cells expressing WTβIII tubulin (red line) to evaluate cellular viability. Each point is the mean value for two independent experiments. These results indicate that expressing βIII tubulin does not result in HeLa cells becoming resistant to L1, while it does result in HeLa cells becoming resistant to paclitaxel.

The identification of new microtubule-targeting agents that can circumvent clinically relevant mechanisms that result in drug resistance could be useful for cancer chemotherapy. The fact that colchicine-binding site agents are known to be less susceptible to multidrug resistance mechanisms that limit the efficacy of many other tubulin inhibitors [22] led us to test the ability of compound L1 to overcome drug resistance. One of the main mechanisms of drug resistance is due to the overexpression of class III β tubulin. This tubulin isotype is found primarily in the brain and alterations in their expression are linked to acquired drug resistance [23,24]. The ability of L1 to overcome β III tubulin-mediated multidrug resistance was evaluated in two HeLa isogenic cell lines [23]. Normal Hela cells and a HeLa cell line overexpressing wild-type β III tubulin (WTβIII) were treated with L1 and paclitaxel as a control, and cell viability was measured. Cell viability was inhibited to a similar extent by L1 in both the WTβIII and parental HeLa cell lines. By contrast, the WTβIII HeLa cell line became more resistant to paclitaxel (IC_50_ 5.6 nM) compared with the parental HeLa cell line (IC_50_ 1.9 nM) (figure 7). These results indicate that the expression of β III tubulin does not result in HeLa cells becoming resistant to L1.

### 2.6. Compound L1 inhibits breast cancer cell lines but not a normal cell line

**Figure 8.**
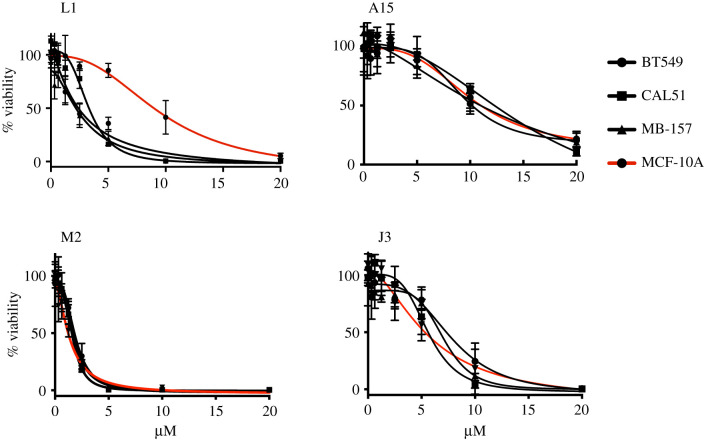
Dose–response curves in three cancer cell lines (BT549, CAL51 and MB-157) are shown in black and non-cancer cell line; MCF-10A is shown in red. They were subjected to a dose–response matrix of the tubulin inhibitor, L1, A15, M2 and J3, respectively. Viability relative to the vehicle control was measured 3 days after treatment. Mean ± s.e.m. for *n* = 3 experiments is shown.

We next investigated whether the four most potent microtubular inhibitors in the *in vitro* assay had specificity against human breast cancer cell lines. The sensitivity of the inhibitors L1, J3, A15 and M2 were evaluated in three breast cancer cell lines, (BT549, CAL51 and MB-157) and a non-cancer cell line from the human mammary gland, MCF-10A (figure 8). Drug sensitivity was determined by culturing breast cancer cell lines (black lines) and the non-cancer cell line (red line) with a range of concentrations of each compound, and viability was measured after 4 days of treatment in triplicate. As shown in figure 8, the IC_50_ of four of these compounds, A15 (9.2–15. µM), M2 (1.4–1.9 µM) and J3 (0.6–0.9 µM), were comparable between the breast cell lines and the non-cancer cell line. However, the IC_50_ of L1 was more than threefold higher in the non-cancer cell line MCF-10A cells (9.6 µM) compared with the three breast cancer cell lines (2.4 to 3.1 µM).

**Figure 9.**
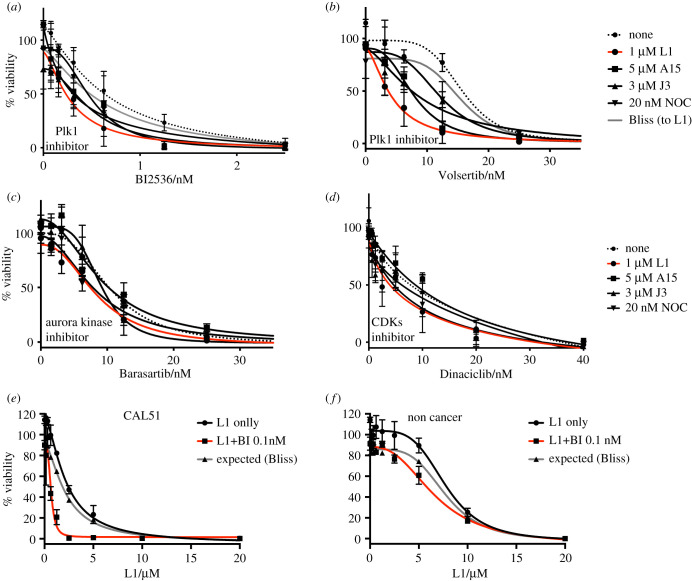
Synergy between tubulin inhibitors and mitotic kinases inhibitors was determined by culturing CAL51 cells with various doses of BI2536 (*a*), Volsertib (*b*), Barasartib (*c*), Dinaciclib (*d*) in the absence (dotted) or presence of tubulin inhibitors, 1 µM L1 (red), 5 µM A15 (black, square), 3 µM J3 (black, triangle) or 20 nM Nocodazole (black, op triangle) added at the time of drug. Expected viability (in A, B, E, F) to L1 according to the Bliss independence model of drug additivity is shown in grey. Mean Relative viability was determined after 4 days after drug addition. CAL51 (*e*) and non-cancer cell line MCF-10A (*f*) were treated with the indicated doses of L1 in the presence (red line) or absence (black line) of 0.1 nM BI2536, added at the time of drug dosing. Mean ± s.e.m. (*n* = 3) experiments is shown.

It has been reported that a microtubule-depolymerizing drug TH588 exhibits a synergistic effect with BI2536, an inhibitor against the mitotic kinase Plk1, on the growth of tissue culture cells. This effect is preferentially increased in cancer cells [25]. Given this synergistic effect, we decided to investigate how a breast cancer cell line and a non-cancer cell line responded to a combination of the four potent anti-microtubular drugs together with inhibitors of the mitotic kinases—Plk1, Aurora kinase and cyclin-dependent kinases (CDKs). To assess synergy, breast cancer CAL51 cells were treated with various doses of mitotic kinase inhibitors in the absence (dotted line) or presence of each of our new tubulin inhibitors at a dose that gives a 5–15% decrease in viability when that compound is used as a single agent. The expected cell viability was calculated according to the Bliss independence model of drug additivity [26,27]. All compounds we examined showed some decreases in viability beyond the expected additive values when combined with inhibitors of Plk1 (BI2536 and Volasertib) (figure 9*a,b*). These results are consistent with a report that has shown a synergistic effect between tubulin and Plk1 inhibitors [25]. By contrast, the combination with an Aurora B inhibitor (Barasertib) or a CDK inhibitor (Dinaciclib) did not display synergy in the compounds we tested (figure 9*c,d*). As L1 displayed specific toxicity in a breast cancer cell line (figure 8), we further compared the synergistic effects of L1 and the Plk1 inhibitor between a cancer cell line (figure 9*e*) and a non-cancer cell line (figure 9*f*), with various doses of L1 in the absence (black) or presence (red) of 0.1 nM BI2536. In the cancer cell line, the expected IC_50_ by the Bliss independence model is 2.1 µM, while the actual IC_50_ is 0.6 µΜ. In the non-cancer cell line, the expected IC_50_ is 7.7 µM and the actual IC_50_ is 6.7 µM. Thus, the synergy between L1 and Plk1 inhibitor is more effective in the breast cancer cell line CAL51 than in the non-cancer cell line MCF-10A.

**Figure 10.**
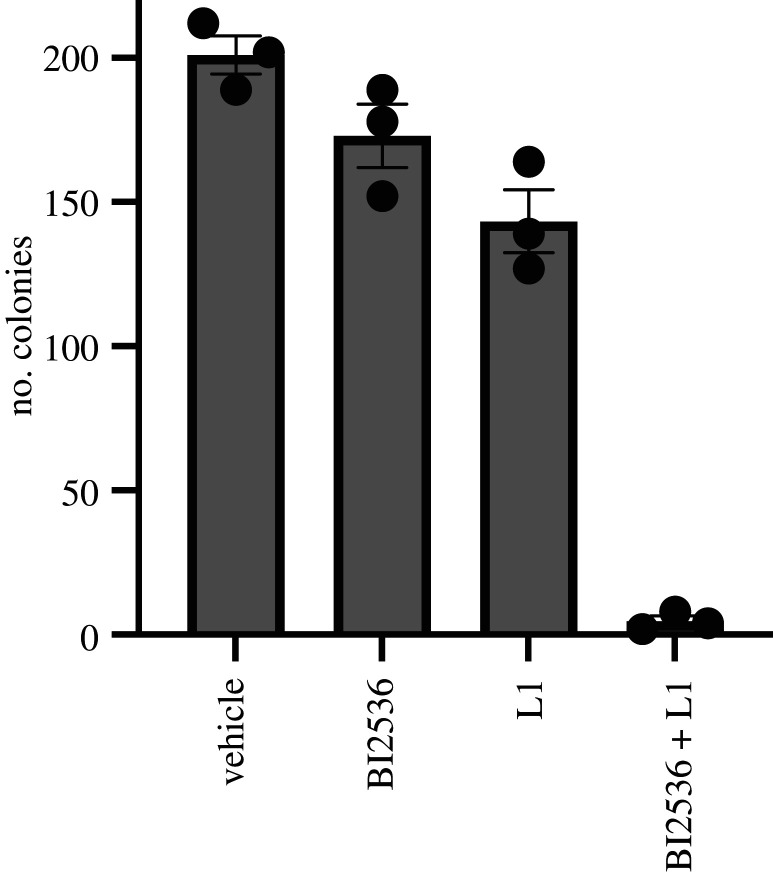
Quantification of the colony formation experiments (*n* = 3) after a 12 h exposure. CAL51 cells were treated with vehicle control, 0.1 nM BI2536, 0.5 µM L1 or 0.1 nM BI2536 and 0.5 µM L1 for 12 h. Cells were grown for 9 days in fresh media after 12 h exposure. Mean ± s.e.m. (*n* = 3) is shown.

Finally, a clonogenic assay was used to further evaluate the synergy effects between the L1 and Plk1 inhibitors to inhibit colony formation of breast cancer CAL51 cells after a 12 h drug exposure. The percentages of colony formation of cells treated with 0.1 nM BI2536 alone and 0.5 µM L1 alone were 89% and 67%, respectively, while cells treated with both 0.1 nM BI2536 and 0.5 µM L1 together were 2% (figure 10) indicating strong synergetic effects in colony formation.

## 3. Discussion

Most microtubule inhibitors are natural products or their derivatives. We have explored here a diverse small-molecule chemical library to identify new microtubule inhibitors in a chemically unbiased manner. Unlike most previous small molecule screens, which identified tubulin inhibitors through their cytotoxic effects on human tissue culture cells, we first employed fission yeast for rapid and cost-effective primary screening followed by screening in human tissue culture cells. This approach was effective in the identification of small molecules which disrupt microtubules in both fission yeast and human cells.

Among the 11 compounds that inhibit microtubule assembly, three of them (L1, K1, E2) contained benzodioxole, which has been found in several known microtubule drugs that target colchicin-binding sites [28,29], including polygamain [19], combretastatin A-2 [30], podophyllotoxin [31], steganacin [32] and TUB091/TUB092 [33]. The crystal structure of *α*/β-tubulin-RB3-tubulin tyrosine ligase (TTL) complex soaked with TUB092 showed that the benzodioxole group positions at the hydrophobic pocket of β-tubulin, a position which is similar to the site that the trimethoxyphenyl moiety (A-ring) of colchicine also occupies [33]. Molecular dynamic modelling of polygamin also showed that the benzodioxole group is buried in this hydrophobic pocket [19]. Since L1 potently competes with colchicine (figure 4), we speculate that the benzodioxole group of L1 also occupies the hydrophobic pocket of β-tubulin. However, in the crystal structure of the *α*/β-tubulin-RB3-TTL complex with podophyllotoxin or its analogue, which have both trimethoxyphenyl and benzodioxole moieties, the position of the benzodioxole matches to the methoxytropolone C ring of colchicine [34], not the aforementioned hydrophobic pocket, suggesting that benzodioxole can fit to the colchicine-binding sites in multiple configurations. Thus, benzodioxole is a versatile chemical group for targeting the colchicine-binding site of β-tubulin.

Microtubule inhibitors such as taxanes and vincristine are commonly used in chemotherapy for breast cancers, ovarian cancers, acute leukaemia malignant lymphoma, and non-small cell lung cancers [35–37]. Our data have revealed that L1 is effective in reducing cell viability in three breast cancer cell lines and that there are significant synergistic effects between L1 and the Plk1 inhibitor, BI2536. This was similar to the reported synergistic effect between BI2536 and TH588, an anti-mitotic compound targeting the colchicine-binding site of β-tubulin [25] as well as 2-hydroxy-dATP diphosphatase MTH1. Combined treatment of BI2536 and TH588 causes mitotic arrest, followed by cell death [25]. These cell deaths depend on the SAC, suggesting that long mitotic arrest is critical for cell death. Tubulin inhibitors can affect the non-mitotic functionality of microtubules, but the combined application of a tubulin inhibitor and mitotic inhibitor can cause mitosis-dependent cell death. In contrast with inhibitors of Aurora B and Cdk1 [38,39], Plk1 has roles in centrosome maturation [40], kinetochore-microtubule stabilization [41] and APC/C activation [42], which may explain why combinatorial inhibition of microtubule assembly and Plk1 but not of Cdk or Aurora B prolongs mitotic arrest, even though Plk1 has also been implicated in correcting erroneous kinetochore-microtubule attachment [43–45] and maintaining checkpoint arrest [46,47]. However, it remains unclear why cancer cell lines are more sensitive to the combinatory inhibitory effects of L1 (or TH588) and BI2536. Patterson *et al*. [25] found such drug synergy in most cancer cell lines among a panel of 29 that they tested. Given that CAL51 exhibited synergistic drug sensitivity to L1, and that BI2536 is a near-diploid chromosomally stable cell line, it seems unlikely that cells with compromised spindle integrity are particularly sensitive to this treatment. In addition, cancer cell specificity of L1 seems unrelated to *in vitro* IC_50_ or cellular IC_50_ of anti-tubulin effects (figures 5 and 8), ruling out the possibility that the drug sensitivity merely reflects cell growth rate. It is also possible that L1 has a secondary target in addition to tubulin in breast cancer cell lines. Further investigations are required to dissect the mechanistic basis for the cancer cell specificity of L1.

### 3.1. Opening Up

The development of drug resistance in cancer is a significant challenge for microtubule inhibitors during their clinical use. For example, the overexpression of βIII tubulin is a prominent cause for taxane resistance in ovarian [48] and breast cancers [49,50]. In the case of L1, however, colchicine and other colchicine-binding site drugs remain effective in cells overexpressing *β*III tubulin [51], and the colchicine-binding site microtubule inhibitors can target tumour vasculature. Therefore, besides being used as anti-mitotic agents, colchicine-binding site compounds can also rapidly depolymerize microtubules of newly formed blood vessels or disrupt existing microvessels to shut down blood supply to tumours [52–54]. While it has been thought that colchicine is too toxic for cancer therapy, benzodioxole-containing compounds such as LI, which target colchicine-binding sites, may offer an anti-microtubule therapeutic strategy with reduced side effects, when combined with other drugs such as anti-Plk1 inhibitors.

Among the compounds that exhibited microtubule assembly defects in fission yeast, L8 and B8 did not show any detectable effect on *in vitro* microtubule polymerization (electronic supplementary material, table S1). As B8 also exhibits mitotic defects in HeLa cells, B8 is a candidate for modulating microtubule dynamics in mitosis without directly binding to tubulins.

This study also identified a panel of compounds that caused growth inhibition of drug-sensitive MDR-sup fission yeast cells, but did not exhibit obvious effects on mitosis in HeLa cells. Although further analyses are required to test if they inhibit pathogenic yeasts and fungi, drugs that selectively inhibit fungi but not human cell lines would be potential candidates for fungicides and antifungal drugs, possibly with potential for agriculture and therapeutic applications [55,56].

## 4. Material and methods

### 4.1. Compounds and chemical reagents

The screening libraries comprised 10 371 compounds from the Rockefeller University High-Throughput Screening Resource Center (http://www.rockefeller.edu/htsrc/libraries). They were resuspended in DMSO (Sigma-Aldrich), and stock solutions were stored at −20°C.

NOC and colchicine were purchased from Sigma-Aldrich, paclitaxel was purchased from Cytoskelton Inc., BI2536, Volsertib, Barasartib and Dinaciclib were purchased from Selleck Chemicals. All chemicals were dissolved in DMSO, kept at −20°C, and used in a 0.25–1% DMSO solution.

### 4.2. Yeast strain, growth conditions and media

The *Schizosaccharomyces pombe* SAK933 strain (h90 ade6 leu1 ura4-D18 GFP-atb2≪kanr sid4-mcherry≪hygr caf5::bsdR pap1Δ pmd1Δ mfs1Δ bfr1Δ dnf2 Δ erg5::ura4+) [57] used for the phenotypic screening in this study was grown at 30°C in yeast extract (YE) medium containing adenine, leucine, uridine and histidine. A haploid wild-type strain 972 h^-^ and its derivative strains were used in the electronic supplementary material, figure S3: *nda2-km52*, *nda3-km311* [58], *cdc25-22* [59,60], *cdc14-208* [61] and *act1-48* [62] spotted onto YE medium containing the same four supplements above and incubated for 3 days at 30°C.

### 4.3. Cell lines

All cell lines were incubated at 37°C in a humidified incubator containing 5% CO_2_ maintained in log phase growth and were routinely monitored for mycoplasma by PCR (Universal Mycoplasma Detection Kit, ATCC). HeLa cells stably expressing H2B-mcherry and mEGFP- *α*-tubulin (a gift of D. W. Gerlich) [63], HeLa WT*β*III cell line (gifts from S. L. Mooberry) [23], MCF-10A (a gift of S. Tavazoie), BT549, CAL51 and MDA-MB-157 cell lines(gifts of H. Funabiki) [15] were grown in DMEM (Thermo Fisher Scientific) supplemented with 10% tet-tested FBS (Atlanta Biologicals) and penicillin-streptomycin (100 u ml^−1^, GIBCO).

For live-cell imaging experiments, 200 000 cells were seeded onto glass-bottom culture dishes (MatTek P35GC-1.5-14-C). Cells were grown overnight before the addition of the compound, and the culture media was replaced with fresh media lacking phenol red to limit phototoxicity before imaging.

### 4.4. Time-lapse microscopy

Live-cell imaging was performed in a LCV110 U VivaView FL incubator microscope (Olympus) equipped with an X-Cite-exacte illumination source (Excelitas Technologies) and Orca-R2 CCD camera (Hamamatsu Photonics). For screening, images were acquired with a 20 × objective every 15 min for 24–48 h in the differential interference contrast (DIC), m-cherry and GFP channels. For assessment of apoptosis, the cells were monitored every 15 min for 24 h. Individual cells were manually analysed using the CellCognition browser [63].

### 4.5. Assessment of apoptosis

Assessment of apoptosis IncuCyte Caspase-3/7 Green apoptosis Reagent from Essen Bioscience (cat. no. 4440) was used to quantitatively evaluate apoptosis in HeLa cells expressing H2B-mcherry and EGFP-α-tubulin cells. Cells were seeded for 24 h onto glass-bottom culture dishes and apoptosis was assessed after each compound was added. The cells were monitored by live microscopy every 15 min for 24 h using DIC microscopy, m-cherry and GFP channels.

### 4.6. *In vitro* tubulin polymerization assay

The Tubulin Polymerization HTS Assay Kit (Cytoskeleton, BK011P) was used according to the manufacturer's instructions. All components were added into a 96-well microtiter plate (Corning Costar, cat. no. 3686), then the tubulin reaction mixture was quickly added to the wells, and tubulin polymerization was initiated and monitored every 1 min at 37°C for 1 h by recording fluorescence of excitation wavelength at 340 nm and emission at 450 nm. The tubulin reaction mixture was composed of 80 mM PIPES (pH 6.9), and 1 mM MgCl2, 1 mM EGTA, 1 mM GTP and 2 mg ml^−1^ of highly purified porcine brain tubulin heterodimer (Cytoskeleton, cat. no. T240).

### 4.7. Differential scanning fluorimetry assay

Thermal shift assays were carried out using an RT-PCR machine (Thermo Fisher, QuantStudio 12 K flex) and SYPRO orange (Invitrogen, cat. no. S6650) as a fluorescence probe. The 25 µl of reaction mixture containing 5 µM porcine brain tubulin (Cytoskeleton, cat. no. HTS03) and 1 : 1000 diluted SYPRO orange dye in general tubulin buffer (80 mM PIPES pH 6.9, 2 mM MgCl_2_ and 0.5 mM EGTA) was incubated and monitored in a RT-PCR instruments using the following program: an initial 2 min hold at 25°C, ramping up in increments of 1°C to a final temperature of 95°C. The Tm values were obtained from the midpoint of the transition.

### 4.8. Colchicine displacement assay

Colchicine displacement assays were performed with 2 µM purified tubulin (Cytoskeleton, Inc) in tubulin reaction mixture as above and displacement assays were performed with 2 µM colchicine or 2 µM colchicine with 0–50 µM L1 using a fluorescence spectrophotometer at 37°C for 2 h. Reaction mixtures were excited at 380 nm and the emission was measured at 438 nm [21,64]. Values were exported as Excel files and graphs were generated in Prism V9 GraphPad.

### 4.9. Measurements of viability and drug combination synergy

Cells were plated in 384-well plates at a density of 800 cells per well in 22.5 µl of media. After 24 h, 2.5 µl drug was diluted in media and was added to each well. Following 5 days of incubation, viability was assessed using CellTiter-Glo (Promega) according to the manufacture's protocol. Luminescence in each well was measured using a Synergy Neo2 plate reader (BioTek).

The Bliss independence algorithm was used to quantify the drug synergy in which drug combinations are compared to sensitivity to individual drugs at the same dose [27].

### 4.10. Colony-forming assay

CAL51 cells were plated at a density of 200 cells per 60 mm tissue culture dish. After 24 h, they were treated with vehicle (DMSO), 0.1 nM BI2538, 0.5 µM L1 or the combination of 0.1 nM BI2538 and 0.5 µM L1, respectively, for 12 h. After an exposure, the cells were washed with warm sterile PBS, and incubated for additional 9 days in fresh media. After the incubation, cells were fixed and stained with a 20% MeOH: 0.01% crystal violet solution for 30 min. Colonies were counted using a stereomicroscope in three independent experiments.

## Data accessibility

This article has no additional data.
